# Retroperitoneal schwannoma preoperatively diagnosed as liver metastasis from colon cancer: A case report

**DOI:** 10.1016/j.ijscr.2019.09.031

**Published:** 2019-09-24

**Authors:** Huanlin Wang, Tomoharu Yoshizumi, Shinji Itoh, Toru Ikegami, Noboru Harada, Yoshinao Oda, Masaki Mori

**Affiliations:** a Department of Surgery and Science, Graduate School of Medical Sciences, Kyushu University, Fukuoka, Japan; b Department of Anatomic Pathology, Graduate School of Medical Sciences, Kyushu University, Fukuoka, Japan

**Keywords:** CT, computed tomography, XELOX, capecitabine plus oxaliplatin, MRI, magnetic resonance imaging, FDG, fludeoxyglucose F 18, PET-CT, positron emission tomography-computed tomography, SUVmax, maximum standard uptake value, Retroperitoneal schwannoma, Liver metastasis, Preoperative diagnosis

## Abstract

**Background:**

Retroperitoneal schwannomas are rare.

**Case presentation:**

We here report a case of 64-year-old woman who was referred to her local hospital for abdominal pain and found to have a palpable tumor. Computed tomography (CT) and colonoscopy revealed a combination of liver and colon lesions and colon cancer with a large liver metastasis was suspected. After neoadjuvant chemotherapy had proved ineffective for her presumed liver metastasis, the patient was referred to our hospital where we performed a simultaneous right hemicolectomy and extended left hepatic lobectomy. The pathological diagnoses were a colonic adenocarcinoma and retroperitoneal schwannoma immediately adjacent to the liver.

**Conclusions:**

Although liver metastasis should be the first provisional diagnosis in patients with advanced colon cancer, retroperitoneal schwannoma should also be suspected in the differential diagnosis of possible liver lesions.

## Highlights

• Retroperitoneal schwannomas are very rare and difficult to make a definite diagnosis.
• This is the first report of surgery for colon cancer and retroperitoneal schwannoma performed simultaneously.
• Although liver lesions accompanied by advanced malignant tumor should be firstly considered as liver metastasis, other rare tumors are also occasionally seen.

## 1 Background

Schwannomas, which originate in Schwann cells of the peripheral nerves, mostly occur in the cephalocervical region and limbs and rarely in the retroperitoneal region. Herein, we report a case of retroperitoneal schwannoma preoperatively diagnosed as a liver metastasis from colon cancer for which the patient underwent a simultaneous left hepatic lobectomy and right hemicolectomy. We also present a review of relevant published reports.

This work has been reported in line with the SCARE criteria [1].

## 2 Case presentation

A 64-year-old woman with no significant medical history was admitted to her local hospital because of right lower abdominal pain and was found to have a palpable tumor. Laboratory tests showed severe anemia (hemoglobin concentration 6.5 g/dL) and high serum concentrations of tumor markers (carcinoembryonic antigen 6.9 ng/mL, cancer antigen 19-9 81 U/mL). Other routine biochemical profile variables were within normal limits.

**Fig. 1:**
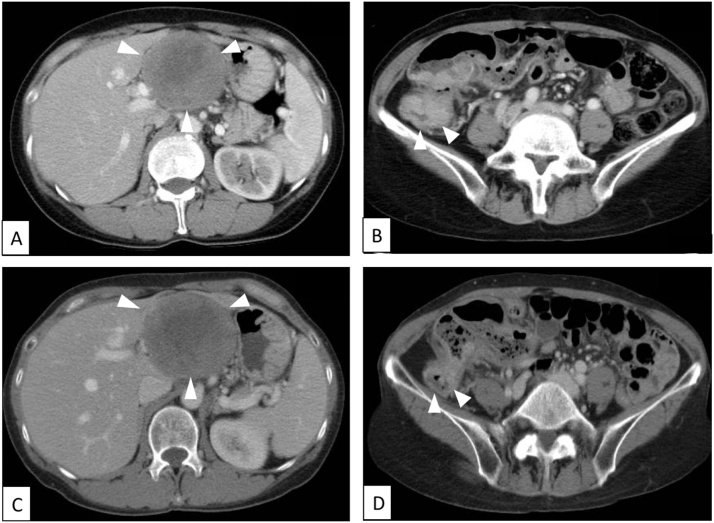
CT images before (**A**, **B**) and after (**C**, **D**) chemotherapy. A 7.0 × 6.0 cm solid tumor apparently located in the left lobe of liver (**A**, **C**) and luminal narrowing with marked wall thickening involving the ascending colon (**B**, **D**) are seen. The colon lesion has become smaller with chemotherapy but the size of the liver lesion has not changed significantly.

**Fig. 2:**
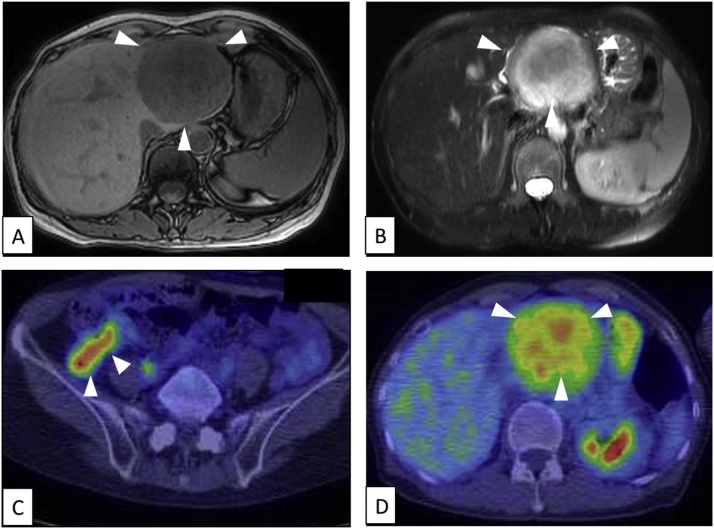
Preoperative MRI and FDG PET/CT scan findings. (**A**) T1-weighted MRI image showing low intensity. (**B**) T2-weighted MRI image showing heterogeneous high intensity. (**C**, **D**) FDG PET/CT scan image showing increased tracer accumulation in both the colon (SUVmax = 5.66) and liver (SUVmax = 5.37) lesions.

**Fig. 3:**
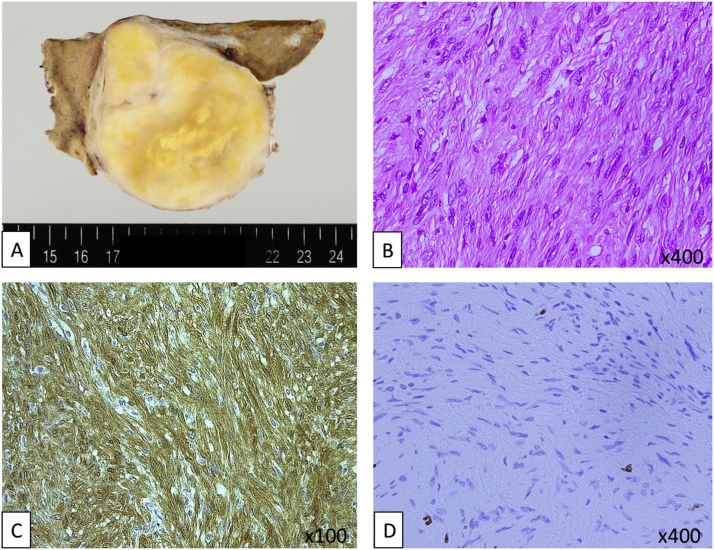
Pathological findings of the liver lesion. (**A**) Macroscopically the 7.0 × 6.0 cm tumor is solitary, yellowish, encapsulated, and has a smooth surface. (**B**) Microscopically, there are proliferating spindle-like tumor cells arranged in a fascicular fashion (**C**) Immunohistochemically, the tumor cells ae positive for S-100 protein. (**D**) The Ki-67 index is about 3%.

Computed tomography (CT) revealed a 7.0 × 6.0 cm solid tumor apparently located in the left lobe of the liver (Fig. 1a) and luminal narrowing and marked wall thickening involving the ascending colon (Fig. 1B). Colonoscopy showed an elevated lesion suggesting advanced colon cancer. Taken together, colon cancer with simultaneous liver metastasis was suspected. Eight courses of neoadjuvant chemotherapy with capecitabine plus oxaliplatin (XELOX) together with bevacizumab were administered. The patient requested surgery and was referred to our hospital. A repeat CT revealed reduction in size of the colonic lesion but no significant change in the liver lesion (Fig. 1C, D) compared with the CT findings before chemotherapy (Fig. 1 A, B). Magnetic resonance imaging (MRI) revealed low intensity on T1-weighted images (Fig. 2A) and heterogeneously high intensity on T2-weighted images (Fig. 2B). Whole body positron emission tomography–computed tomography (PET/CT) imaging showed increased tracer accumulation in both the colon (maximum standard uptake value [SUVmax] = 5.66) (Fig. 2B C) and liver (SUVmax = 5.37) (Fig. 2D) lesions. Since no other distant metastasis besides the liver metastasis was observed, simultaneous right hemicolectomy and extended left hepatic lobectomy were performed. The operative time was 5 h and 3 min and the blood loss 562 mL. Pathological examination of the resected specimen of colon showed proliferating well-differentiated adenocarcinoma cells with massive mucinous degeneration accompanied by lymph node metastasis. Macroscopically, the liver specimen contained a solitary, yellowish encapsulated 7.0 × 6.0 cm tumor with a smooth surface that was adjacent to rather than within the liver (Fig. 3A). On microscopic examination, proliferating spindle-like tumor cells arranged in a fascicular fashion and accompanied by collagenous fibers were observed (Fig. 3B). Immunohistochemically, the tumor cells were strongly positive for S-100 protein (Fig. 3C) and the Ki67-index was about 3% (Fig. 3D). These features resulted in a diagnosis of retroperitoneal schwannoma with no definite evidence of malignancy.

## 3 Discussion

Schwannomas, which originate in Schwann cells in nerve sheaths, are slowly growing tumors that mostly occur in peripheral nerves in the skin and subcutaneous tissue of the head and neck; however, they can originate in other organs such as the gastrointestinal tract [2] and bone. Previous studies have shown that retroperitoneal schwannomas are rare, comprising 0.7–2.7% of all schwannomas [3,4]. Li et al. reported an analysis of 82 case of retroperitoneal schwannoma and found no predisposition regarding sex (men: 46%, women: 54%).

Although most cases are asymptomatic and detected incidentally by physical examination, abdominal symptoms such as abdominal distension and pain can occur if the tumor is large and compressing adjacent organs. Strauss et al. reported 28 cases of benign retroperitoneal schwannomas from a single center, 13 of whom were asymptomatic whereas eight patients presented with abdominal pain and seven with a palpable mass [5]. The case we have reported here also presented with abdominal pain and a palpable mass; however, the simultaneous presence of an advanced colon cancer made it difficult to determine the cause of the abdominal pain.

On pathological examination, schwannomas can be categorized into two subtypes: Antoni A and Antoni B. The majority are of Antoni A type, which is characterized by compact spindle cells arranged a palisading fashion, whereas a minority are of Antoni B type, which is characterized by collections of lipid-laden histiocytes and thick-walled, hyalinized blood vessels. Immunohistochemical staining is a useful tool to help to make a diagnosis of schwannoma because most cases have strong diffuse staining for S100 protein in the cell nuclei and cytoplasm [6,7]. In our case, the microscopic findings were of proliferating compact spindle-cells arranged in a palisading fashion, indicating it was of Antoni A type. Although some bizarre cells were observed, immunohistochemistry was strongly positive for S-100 protein and the Ki67 index was about 3%. Thus, the final pathological diagnosis was benign schwannoma of Antoni A type.

Because there are no standards means of diagnosis, a preoperative diagnosis of retroperitoneal schwannoma is always difficult to make. CT scans typically reveal a low-density mass [8], whereas on MRI hypointensity on T1-weighted images and hyperintensity on T2-weighted images are typical of schwannoma [[9], [10], [11]]. M. Yamamoto et al. reported a case of primary hepatic schwannoma and summarized the typical radiological features of hepatic schwannoma [12]. Our case showed a hypointensity on T1-weighted images and hyperintensity on T2-weighted images, which is consistent with those of typical schwannomas. Fludeoxyglucose F 18 (FDG) PET/CT is also useful for diagnosing peripheral nerve sheath tumors such as schwannomas, especially for distinguishing whether the tumor is malignant or benign [13]. However, in our case the FDG maximum uptake values for the colon and liver lesion were very similar (SUVmax = 5.66 vs. SUVmax = 5.37), which made the preoperative diagnosis difficult. Angiography to assess the vascular supply of the tumor may also help in diagnosis of retroperitoneal schwannoma, especially when planning surgery [14]. However, because angiography is invasive and carries a risk of hemorrhage, it is not widely performed. The presence of our patient’s simultaneous advanced colon cancer and our difficulty in distinguishing whether the apparent hepatic tumor was located in the left hepatic lobe or outside the liver resulted in misdiagnosis of the tumor as a liver metastasis from colon cancer. On pathological examination it was found to be a retroperitoneal schwannoma located outside the liver and with no evidence of liver parenchyma invasion.

## 4 Conclusions

We here present a rare case of retroperitoneal schwannomas which was difficult to diagnose preoperatively. Although liver metastasis should be the first provisional diagnosis when the patient has an advanced colon cancer, retroperitoneal schwannoma should also be suspected in the differential diagnosis of possible liver lesions.

## Sources of funding

Huanlin Wang and other participating authors have no involvement as above.

## Ethical approval

This case report is not research study, therefore approval was not given.

The ethical approval has been exempted by our institution.

## Consent

Written informed consent was obtained from the patient for publication of this case report.

## Author’s contribution

TY, SI, TI, and NH performed the operation. TY, SI and NH determined the treatment plan. HW and TY prepared and wrote the manuscript. HW, YO made the pathological diagnosis. MM gave final approval for the version to be published. All authors have read and approved the final manuscript.

## Registration of research studies

The paper is a clinical report, no research involved.

## Guarantor

Huanlin Wang accept full responsibility for this work.

## Provenance and peer review

Not commissioned, externally peer-reviewed.

## Declaration of Competing Interest

All authors have no conflicts of interest.
